# Effect of Face-to-Face and WhatsApp Communication of a Theory-Based Health Education Intervention on Breastfeeding Self-Efficacy (SeBF Intervention): Cluster Randomized Controlled Field Trial

**DOI:** 10.2196/31996

**Published:** 2022-09-14

**Authors:** Farahana Mohamad Pilus, Norliza Ahmad, Nor Afiah Mohd Zulkefli, Nurul Husna Mohd Shukri

**Affiliations:** 1 Department of Community Health Faculty of Medicine and Health Sciences Universiti Putra Malaysia Serdang Malaysia; 2 Department of Nutrition, Faculty of Medicine and Health Sciences Universiti Putra Malaysia Serdang Malaysia

**Keywords:** self-efficacy, breastfeeding, intervention, social cognitive theory

## Abstract

**Background:**

The exclusive breastfeeding rate in Malaysia is still not satisfactory. Previous studies have shown that breastfeeding self-efficacy is one of the determinants of exclusive breastfeeding, and it can be improved using social cognitive theory. WhatsApp, which is widely used among Malaysians, could be leveraged as a platform to deliver health education interventions.

**Objective:**

This study aimed to develop, implement, and evaluate the effect of using a face-to-face and WhatsApp-based health education intervention based on social cognitive theory, namely the Self-Efficacy in Breastfeeding (SeBF) module, on mothers' self-efficacy, knowledge, and attitudes in a district in Selangor state.

**Methods:**

This study was a 2-arm, parallel, single-blind, cluster randomized controlled field trial with an intervention and a control group involving primigravida or multigravida mothers who reside in a district in Selangor state and did not exclusively breastfeed during their previous pregnancy. All 12 maternity and pediatric clinics in this district were randomly divided into 6 intervention and 6 control groups. A total of 172 pregnant mothers were randomly assigned to the intervention group (n=86) or the control group (n=86). The control group received usual routine care. The primary outcome was breastfeeding self-efficacy, while secondary outcomes were knowledge and attitude toward breastfeeding. Each subject was assessed at 4 time points: at baseline, immediately after the intervention, 4 weeks post partum, and 8 weeks post partum. Generalized mixed model analysis was applied to measure the effect of health education on breastfeeding self-efficacy, knowledge, and attitude after the intervention.

**Results:**

The response rate was 81% (139/172), with the dropout rate being 7% (6/86) in the intervention group and 31% (27/86) in the control group. In the intent-to-treat analysis, the intervention group showed a significant increase in the mean total breastfeeding self-efficacy score 8 weeks after delivery compared with the control group (*F*_21,601_=111.73, *P<*.001). In addition, the mean total score for breastfeeding knowledge increased significantly in the intervention group after the intervention compared to the control group (*F*_21,601_=8.33, *P<*.001). However, no significant difference was found in the mean total score for breastfeeding attitude after the intervention (*F*_21,602_=5.50, *P*=.47).

**Conclusions:**

Face-to-face and WhatsApp-based participation in the SeBF program, designed on the basis of social cognitive theory, contributed to improved self-efficacy and knowledge about breastfeeding. Further studies need to be conducted with a longer duration (until 6 months post partum) to evaluate its effectiveness in increasing exclusive breastfeeding. Furthermore, new strategies in health education need to be developed to improve breastfeeding attitudes.

**Trial Registration:**

Thaiclinicaltrials.org TCTR20200213004; https://www.thaiclinicaltrials.org/show/TCTR20200213004

## Introduction

### Background

The overall prevalence of exclusive breastfeeding in Malaysia was 47.1% (95% CI 43.13-51.18) [1], which is below the national target of 70% by 2025 [2]. Previous studies have shown that breastfeeding self-efficacy is one of the determinants of exclusive breastfeeding [3,4]. Breastfeeding self-efficacy refers to a woman's confidence in breastfeeding ability with an infant [3,5]. Several factors are associated with breastfeeding self-efficacy, including support and guidance, experience and stress, postpartum experiences, and social environment [5-7]. Social cognitive theory (SCT) has been shown to be effective in previous breastfeeding self-efficacy intervention studies [8-13]. SCT involves a cognitive dynamic process that assesses individuals' beliefs and ability to engage in healthy behaviors [8].

On the other hand, several methods have been used to deliver knowledge and skills to mothers, including face-to-face conversations, phone calls, and web-based applications [5,10-12,14,15]. WhatsApp, a cross-platform application that works on all major smartphone platforms such as Android, iPhone, and Windows Mobile, has approximately 500 million users worldwide [16]. According to a recent study [17], WhatsApp is preferred by 98.7% of Malaysian respondents, while Facebook Messenger is preferred by 54% of them. Given the widespread use of WhatsApp, we sought to evaluate the effectiveness of its use in providing health education to pregnant women.

### Objectives

This study aimed to determine the effect of an SCT-based intervention called Self-Efficacy in Breastfeeding (SeBF) to improve breastfeeding self-efficacy through face-to-face communication and WhatsApp.

## Methods

### Study Design

This 2-arm, parallel, single-blind cluster randomized controlled field trial, comprising an intervention group and a control group, was conducted at maternal and child health clinics in Hulu Langat district, Selangor, Malaysia. The health clinics are considered a cluster for this study. The intervention group received the SeBF intervention, whereas the control group received standard routine brief counselling by health care personnel about breastfeeding and breastfeeding pamphlets.

### Recruitment and Inclusion Criteria

Pregnant women between 34 and 37 weeks of gestation, who presented for antenatal care at maternal and child health clinics, were offered participation in this study, and their eligibility was assessed. Eligible mothers were primigravida or multigravida women who had not exclusively breastfed during a previous pregnancy and had a cell phone with Internet access and the WhatsApp app. Mothers who were taking medications such as anticancer drugs and those who had medical or pregnancy-related complications that hindered or complicated breastfeeding (heart disease, cancer, nephritis, active or untreated tuberculosis, HIV or AIDS, active breast herpes lesions, and severe malnutrition) were excluded from this study [14]. During recruitment, all participants have been informed that intervention was being offered. Mothers who expressed interest were provided with participant information sheets and informed consent forms.

### Randomization and Allocation Concealment

The cluster comprised 12 maternal and child health clinics in Hulu Langat District. All chosen health clinics were randomly allocated into intervention and control groups by a nurse who was not involved in this study. All selected clinics were labeled, and Stat Trek software was used to perform simple randomization with a 1:1 allocation ratio [15]. The researcher was only aware of the intervention group's assignment after the randomization procedure was completed. Antenatal mothers who met the eligibility requirements were recruited and consented with an equal number of participants assigned to each clinic. Participants were blinded to group assignments. Participants were blinded to the fact that awareness of being part of the control group could influence their responses to the questionnaires.

### Sample Size Calculation

The sample size was estimated on the basis of Lemeshow and Lwanga's Sample Size Determination in Health Studies [18]. The formula for 2 population proportions was used for hypothesis testing purposes. The sample size calculated was on the basis of a 30% increase in breastfeeding self-efficacy in the control group and a 55% increase in breastfeeding self-efficacy in the intervention group [11], with an α of .05 and β of .20, an intraclass correlation coefficient of 0.05 [19], attrition rate of 20% [20], and an average cluster size of 20 with a design effect of 2.45. After adjusting for the clustered design effect, the final minimum sample size required was 160 participants, with 80 participants in the intervention and control groups.

### Intervention Module

The SeBF intervention was a newly developed module to improve breastfeeding self-efficacy among mothers. The development was based on SCT and prior intervention studies [5,8-10,12,15,18]. Breastfeeding self-efficacy was the main aim as it is one of the important determinants for successful exclusive breastfeeding [21]. Based on this intervention's success, it will be further evaluated for exclusive breastfeeding purposes.

This newly developed SeBF module was consulted and discussed with 2 Public Health Medicine Specialists and a Nutrition Specialist. This intervention module applied the SCT constructs such as observational learning, personal experience, verbal persuasion, problem-solving, self-efficacy, and outcome expectation. The SCT constructs used in the SeBF module showed in Multimedia Appendix 1.

The SeBF module was developed to be delivered face to face and through WhatsApp. The intervention consisted of training and reinforcement phases. The training phase involved a face-to-face session of 30 minutes, while the reinforcement phase involved WhatsApp messages weekly until 4 weeks post partum. The WhatsApp messages were sent every Monday at 2:30 PM for 15 minutes. The WhatsApp group function was used to distribute information, concerns, and issues; clarify any misunderstandings about breastfeeding practice; and provide a reminder to all participants.

The privacy and confidentiality of the participants were protected via a private group formation on WhatsApp. Thus, no other person apart from those recruited by the researcher could have access to the group. In addition, no personal information was exposed in the WhatsApp group. This module has been pilot-tested among 30 antenatal mothers not included in the main study. The SeBF intervention was delivered by a researcher who is also a medical doctor.

### Study Instruments

The primary outcome of this intervention study was self-efficacy in breastfeeding scores, and the secondary outcome represents scores on knowledge and attitudes toward breastfeeding. These outcomes were assessed in the intervention and control groups immediately post intervention, 4 weeks post partum, and 8 weeks post partum.

### Breastfeeding Self-efficacy

The 13-item Breastfeeding Self-Efficacy Scale–Short Form was used to assess mothers' confidence in their ability to successfully breastfeed their infant [22,23]. All items are preceded by the phrase “I can always” and are anchored on a 5-point Likert scale, where 1=“not at all confident” and 5=“always confident” [10]. Each item is presented positively, and the sum of the scores gives a range of 14 to 65, with higher scores indicating greater breastfeeding self-efficacy [10]. The Cronbach α coefficient was .90 [24].

### Knowledge on Breastfeeding

A validated questionnaire with 47 items was used to assess knowledge about breastfeeding. These included general knowledge, benefits to mothers and babies, effective feeding method, duration of breastfeeding, expressed breast milk (EBM), storage of EBM, complementary foods, and problems with breastfeeding [25]. Each item had categorical responses of “True,” “False,” or “Not sure.” A correct answer was scored as “1,” while a wrong and an unsure answer were scored as “0.” The total knowledge score ranged from “0” to “47,” with higher scores representing more knowledge. The internal consistency of Cronbach α was .70 [25].

### Breastfeeding Attitude

The 16-item Iowa Infant Feeding Attitude Scale was used to assess mothers' attitudes toward breastfeeding [26,27]. Mothers were asked to indicate the extent to which they agreed with each statement on a 5-point Likert scale ranging from 1=“strongly disagree” to 5=“strongly agree.” Items endorsing formula feeding were reverse-scored (1=5, 2=4, 3=3, 4=2, and 5=1), and an overall attitude score was calculated from the equally weighted sum of responses to each item. The total attitude scores ranged from “16” for a positive attitude toward formula breastfeeding to a maximum score of “80” for an attitude favoring breastfeeding. The Cronbach α for this instrument was ≥.85 [28].

### Data Collection

The data collection for this study was conducted from January to December 2020. Baseline data were collected after participants' recruitment. A second assessment was conducted immediately after the training phase, followed by 4 and 8 weeks post partum. Concurrently, data from the control group were also collected at the same 4 time points. Attendance at the maternity and child health clinic was severely affected by the pandemic COVID-19. For the 4- and 8-week postpartum follow-ups, data collection was switched from hard copy self-administered questionnaires to Google Forms.

### Statistical Analysis

SPSS (version 25; IBM Corp) was used for the analyses. Shapiro-Wilk, Komolgorov-Smirnov, and histogram tests were used to determine normal distribution. In the descriptive study, data were presented as mean, SD, frequency, and percentage values. The Pearson chi-square or Fisher exact test was used to determine the homogeneity of baseline data between the intervention and control groups for categorical data, and the Student *t* test was used for continuous data. The effectiveness of the intervention was determined using generalized mixed model analysis (GLMM), which controlled for baseline data covariates such as age, ethnicity, education level, maternal employment, and monthly family income. A significance level of .05 with a 95% CI was used for the study. Thus, the null hypothesis with a *P* value of >.05 was rejected.

### Ethics Approval

The National Medical Research Registry granted ethical approval for this study (NMRR-19-2712-50586). Each respondent provided written and informed consent during the data collection process. All participants’ information was kept strictly confidential. The study was prospectively registered in the Thai Clinical Trial Registry with identification number TCTR20200213004.

## Results

### Response Rate

**Figure 1:**
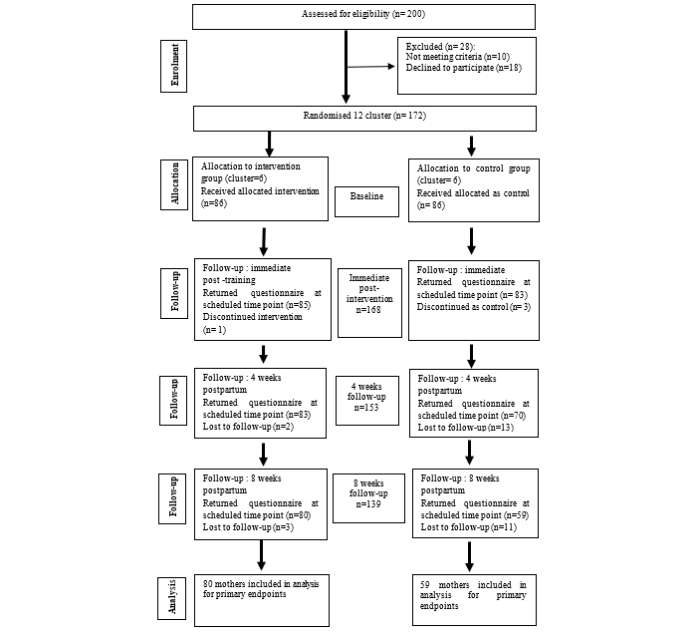
Sample recruitment and dropouts throughout the study period.

A total of 139 mothers completed all 4 points of follow-up, resulting in a response rate of 81% (139/172) 8 weeks after delivery. The dropout rate was 31% (27/86) in the control group and 7% (6/86) in the intervention group. Figure 1 summarizes the final research schedule based on the CONSORT (Consolidated Standards of Reporting Trials) statement (Multimedia Appendix 2) [29].

### Baseline Data

Table 1 provides an overview of the baseline data for the respondents. There were no significant differences in categorical or continuous variables between the intervention and control groups (*P*>.05). The household income in Malaysia was categorized as the B40 (below 40% of population) group, the M40 (middle 40% of population) group, and the T20 (top 20% of the population) group [30].

**Table 1:** Distribution of continuous and categorical variables at baseline (n=172).

Variables		Intervention (n=86)	Control (n=86)	Difference between the conditions	
				Fisher exact test or *t* test (*df*)	*P* value
**Mother’s current age (years), n (%)**				N/A^a^	.25
	19-24	24 (28)	18 (21)		
	25-35	57 (66)	66 (77)		
	36-45	5 (6)	2 (2)		
**Ethnicity, n (%)**				N/A	.09
	Malay	73 (85)	75 (87)		
	Chinese	4 (5)	2 (2)		
	Indian	3 (3)	8 (9)		
	Others	6 (7)	1 (1)		
**Highest education level, n (%)**				N/A	.48
	Primary	2 (2)	5 (6)		
	Secondary	28 (33)	25 (29)		
	Higher education	56 (65)	56 (65)		
**Mother’s employment status, n (%)**				N/A	.33
	Unemployed	28 (32)	24 (28)		
	Self-employed	4 (5)	9 (10)		
	Government sector	7 (8)	11 (13)		
	Private sector	47 (55)	42 (49)		
**Total monthly household income (Malaysian Ringgit [RM]; 1 RM=US $0.22)^b^, n (%)**				N/A	.25
	B40 (<RM 4850)	62 (72)	58 (67)		
	M40 (RM 4850-10,959)	23 (27)	23 (27)		
	T20 (>RM 10,960)	1 (1)	5 (6)		
Total score for breastfeeding self-efficacy, mean (SD)		22.44 (6.82)	24.38 (8.61)	–1.63 (169)	.11
Total score for breastfeeding knowledge, mean (SD)		22.44 (6.83)	24.12 (8.27)	–1.45 (169)	.15
Total score for breastfeeding attitudes, mean (SD)		56.27 (7.00)	56.97 (6.69)	–0.67 (169)	.51

^a^N/A: not applicable.

^b^Household income was categorized on the basis of the Department of Statistics Malaysia’s classification of B40, M40, and T20.

### Primary and Secondary Outcomes

Table 2 compares breastfeeding self-efficacy, knowledge about breastfeeding, and attitudes toward breastfeeding between the intervention and control groups at baseline, immediately after training, and 4 and 8 weeks post partum. Bivariate analyses show no significant difference between intervention and control groups for all time points, except for the total score for knowledge 8 weeks post partum.

Table 3 shows the GLMM results for the values of self-efficacy, knowledge, and attitude toward breastfeeding after controlling for covariates. The results show a significant difference between the intervention and control groups for self-efficacy and knowledge of breastfeeding (*F*_21,601_=111.728, *P*<.001 and *F*_21,601_=8.331, *P*<.001, respectively). However, there was no significant difference between the two groups for attitudes toward breastfeeding.

**Figure 2:**
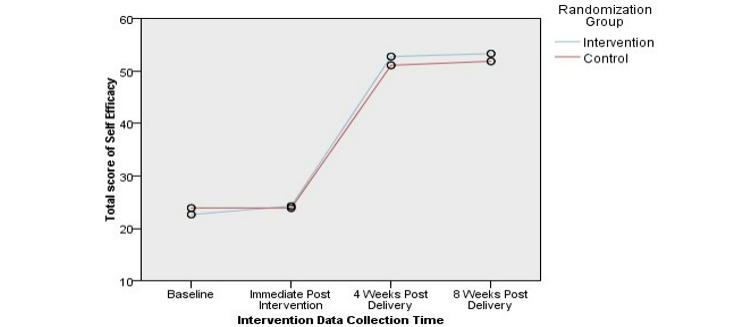
Total scores on self-efficacy, showing the interaction between group and time, for all respondents.

**Figure 3:**
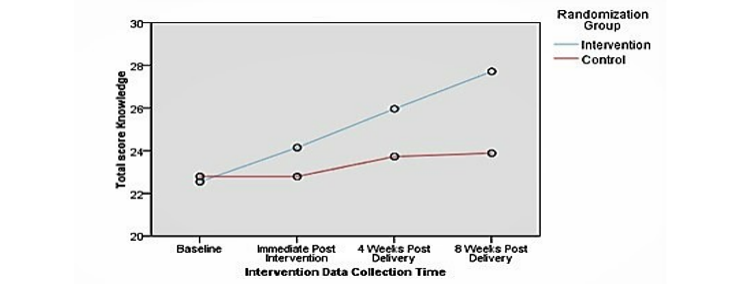
Total scores on breastfeeding knowledge, showing the interaction between group and time, for all respondents.

**Figure 4:**
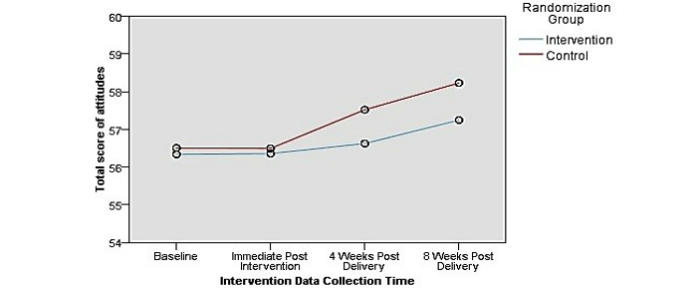
Total scores of attitudes on breastfeeding showing the interaction between group and time, for all respondents.

Figure 2 shows that breastfeeding self-efficacy improved in the intervention group over all 4 time points. The control group had an almost identical pattern to that of the intervention group. Although the control group showed an almost similar trend to the intervention group, it did not reach a high level of self-efficacy at 8 weeks after the intervention. Regarding breastfeeding knowledge, Figure 3 shows that the overall rating of breastfeeding knowledge improved for all respondents in the intervention group compared to that in the control group. Figure 4 shows that attitudes toward breastfeeding did not improve after the intervention study.

**Table 2:** Breastfeeding self-efficacy, knowledge, and attitudes between intervention and control groups at baseline, immediately after the intervention, and 4 and 8 weeks post partum.

Variables		At baseline	Immediately post intervention	4 weeks post partum	8 weeks post partum
**Total score of self-efficacy**					
	Intervention group score, mean (SD)	22.44 (6.82)	24.02 (6.37)	52.60 (9.29)	53.34 (9.16)
	Control group score, mean (SD)	24.38 (8.61)	24.37 (8.55)	51.22 (9.32)	51.89 (8.44)
	*t* test (*df*)	–1.63 (169)	–0.30 (169)	0.95 (161)	0.90 (145)
	*P* value	.11	.76	.34	.37
	95% CI for difference of means	–6.43 to –0.71	–4.28 to 0.41	–1.50 to 4.26	–1.58 to 4.20
**Total score of knowledge**					
	Intervention group score, mean (SD)	22.44 (6.83)	24.02 (6.405)	26.10 (5.66)	27.54 (5.98)
	Control group score, mean (SD)	24.12 (8.27)	24.12 (8.217)	25.11 (7.43)	24.57 (7.41)
	*t* test (*df*)	–1.45 (169)	–0.08 (169)	0.94 (157)	2.54 (141)
	*P* value	.15	.94	.35	.01^a^
	95% CI for difference of means	–3.97 to 0.61	–2.32 to 2.13	–1.09 to 3.08	0.65 to 5.29
**Total score of attitudes**					
	Intervention group score, mean (SD)	56.27 (7.0)	56.26 (6.99)	56.71 (5.53)	57.35 (6.06)
	Control group score, mean (SD)	56.97 (6.7)	56.98 (6.66)	57.91 (7.789)	58.88 (8.56)
	*t* test (*df*)	–0.67 (170)	–0.69 (170)	–1.13 (158)	–1.25 (140)
	*P* value	.50	.49	.27	.24
	95% CI for difference of means	–2.76 to 1.37	–0.72 to 1.04	–3.33 to 0.93	–4.11 to 1.05

^a^Statistically significant at *P*<.05.

**Table 3:** The effect of health education on mothers' overall ratings of self-efficacy, knowledge, and attitudes toward breastfeeding.

Outcomes and parameters		*F* test (*df*)	*P* value^a^
**Total scores of breastfeeding self-efficacy**			
	Group	0.85 (1, 601)	.36
	Time	413.95 (3, 601)	<.001^b^
	Group×time	111.73 (21, 601)	<.001^b^
**Total scores of breastfeeding knowledge**			
	Group	6.38 (1, 601)	.02^b^
	Time	4.29 (3, 601)	.005^b^
	Group×time	8.33 (21, 601)	<.001^b^
**Total scores of breastfeeding attitudes**			
	Group	0.91 (1, 602)	.34
	Time	0.38 (3, 602)	.77
	Group×time	5.50 (21, 602)	.47

^a^Using a generalized linear mixed model adjusted for respondents’ age, ethnicity, level of education, employment, and household income.

^b^Statistically significant at *P*<.05.

## Discussion

### Principal Findings

The purpose of the current study was to develop, implement, and evaluate the effect of the SeBF intervention on breastfeeding self-efficacy among antenatal mothers in Hulu Langat District, Selangor. The module was developed on the basis of SCT and delivered face to face and on WhatsApp. The findings showed that the intervention group had significantly increased their breastfeeding self-efficacy and knowledge scores compared to the control group. These significant changes could be contributed by the use of the SCT, namely observational learning, personal experience, verbal persuasion, and problem-solving to fulfil the end expectation and breastfeeding self-efficacy [31-33]. It included an antenatal period that began at the earliest 34 weeks of pregnancy and a postpartum period that lasted until 8 weeks post partum. It helped ensure that all intervention participants received an adequate and optimal dose of the SeBF intervention during the most critical period, from the third trimester of pregnancy to 8 weeks post partum [32]. Numerous approaches, including educational talk, practical breastfeeding videos, model demonstrations, and group discussions, contributed to respondents’ increased self-efficacy. The usage of mobile technology, specifically WhatsApp, may have improved the primary and secondary outcomes. It facilitated the communication between the researcher and the participants in the intervention group. Hence, any problems faced by the mothers can be solved immediately. WhatsApp was chosen because it is a popular social media platform among Malaysians [30]. Therefore, it is readily used rather than requesting the respondents to install other new applications for this study.

### Comparison With Prior Work

This study’s findings are consistent with those of a previous study conducted in Iraq [13], indicating a significant difference in the change in mean breastfeeding self-efficacy scores between mothers who received the intervention and those who did not receive the intervention by applying SCT in their study. This study found that although all pregnant women in the study had some prenatal visits before enrollment, their breastfeeding knowledge was poor. This suggests the need to educate women about breastfeeding during their routine antenatal appointments [34]. Overall knowledge scores were higher in the intervention group than in the control group. An Iranian study found that women who received antenatal education have significantly higher knowledge scores, which resulted in higher mean breastfeeding self-efficacy scores; 53.98 (SD8.50) in the intervention group and 43.41 (SD 8.12) in the control group (*P*=.001) [13]. Another study in Canada reported the same result, with a significant increase in participants' knowledge scores following the educational breastfeeding intervention (mean knowledge scores of 24.14, SD 4.08 post intervention vs 11.43, SD 4.78 before the intervention, *P*=.001) [35].

There is no significant difference in the mean total score of breastfeeding attitude between intervention and control groups in this study. Our finding is consistent with a study in Jordan, which found that despite an improvement in respondents' self-efficacy for breastfeeding, their attitude ratings remained unchanged [27]. Furthermore, a study conducted in India found that the number of mothers with a favorable attitude dropped from the first to the third follow-up visit, despite improved knowledge at the baseline visit [36]. The most frequently cited reasons for their change in attitude toward breastfeeding were a perception of insufficient milk secretion, concern for the baby's health, concern that the baby would not gain enough weight on breastfeeding alone until 6 months, and complications such as breast engorgement and sore nipples during breastfeeding [36]. Increasing the intensity of WhatsApp communication may help improve the breastfeeding attitude and subsequently increase breastfeeding self-efficacy [11,12].

### Strengths and Limitations

This study was a randomized controlled field trial with a reasonable participation rate and adequate follow-up despite the COVID-19 pandemic during data collection. Randomized assignment to the intervention and control groups makes the two comparable and increases validity [36]. It is the first study to employ innovative teaching and follow-up techniques for breastfeeding intervention using WhatsApp. Modification of educational materials and linking of responses to more accessible and appealing social media platforms are critical components of modern education [37].

However, this study has some limitations. Because of the self-completed questionnaire used in this study, social desirability may have been observed, with individuals responding positively being considered successful breastfeeding mothers. This study may also not be relevant to individuals who do not have access to a smartphone, as the follow-up of the intervention was conducted via WhatsApp. Furthermore, it is difficult to track whether the intervention group read and digested the material distributed on WhatsApp. In the future, the researcher should ask respondents random questions to determine if they understood the intervention material.

Replicating this intervention may be difficult with limited human resources since it will impose an additional demand on health care personnel. While some health care facilities may be able to provide educational talk, group discussions, and WhatsApp group follow-ups, others may find it time consuming and difficult. Before adopting a clinic-level intervention, considerations of appropriateness, time, and human resources are required.

### Conclusions

Participation in the SeBF intervention, a face-to-face and WhatsApp-based intervention using SCT, significantly improved self-efficacy and knowledge about breastfeeding. Nevertheless, this study showed that respondents' attitudes did not improve. This study showed that WhatsApp could be a practical tool in complementing breastfeeding health education to mothers. The user-friendly application can deliver simple and easy-to-read health messages and facilitate communication between health care providers and mothers. In future, the study period should extend to 6 months to examine the module's capability in attaining the exclusive breastfeeding goals. Furthermore, new strategies in health education need to improve breastfeeding attitudes.

## Appendix

Multimedia Appendix 1 Social Cognitive Theory Constructs Used in SeBF Module.

Multimedia Appendix 2 CONSORT eHEALTH Checklist (V1.6.2).

## Abbreviations

EBM: expressed breast milk
GLMM: generalized linear mixed model
SCT: social cognitive theory
SeBF: Self-Efficacy in Breastfeeding
