# Supplementary material for: Effect of Face-to-Face and WhatsApp Communication of a Theory-Based Health Education Intervention on Breastfeeding Self-Efficacy (SeBF Intervention): Cluster Randomized Controlled Field Trial
Source: JMIR Mhealth Uhealth. 2022 Sep 14;10(9):e31996. doi: 10.2196/31996 (PMC9520384; doi:10.2196/31996)
Supplement: Multimedia Appendix 1 [file mhealth_v10i9e31996_app1.doc]

**Multimedia Appendix 1.** Social Cognitive Theory Constructs Used in SeBF Module.

**SOCIAL COGNITIVE THEORY CONSTRUCTS USED IN SEBF MODULE**

| **No.** | **Phase / Timing of Intervention** | **SCT Constructs** | **Content** | **Strategies** |
| --- | --- | --- | --- | --- |
| 1. | Training Phase / Antenatal Phase  (face-to-face)   - 30 minutes | Vicarious experiences  (Observational learning) | - What is breastfeeding - Importance and benefit of breastfeeding  - Motivation and reality of breastfeeding  - Difficulties in breastfeeding  - Common issues in breastfeeding  - Steps to successful breastfeeding | 1) Breastfeeding talk  2) Practical session on breastfeeding practice breastfeeding video  3) Demonstration on breastfeeding positioning and proper latching using model |
|  |  | Performance accomplishment  (Personal experience) | - Personal opinion and idea on breastfeeding  - Previous experience in breastfeeding  - Difficulties in breastfeeding  - Solution on breastfeeding problem  - Motivation on breastfeeding | 4) Group Discussion  - 5 pregnant mothers in each group |
| 2. | Reinforcement/ Postpartum Phase  (week 1 postpartum)  -15 minutes | Verbal persuasion  (Appraisal from others) | - Reminder on importance of breastfeeding  - Reminder on successful steps on breastfeeding  - Guidance | WhatsApp follow up  Breastfeeding Support Group |
|  | Reinforcement/ Postpartum Phase  (week 2 postpartum)  -15 minutes | Problem Solving | - Motivational support  - To answer question on breastfeeding | WhatsApp follow up  Breastfeeding Support Group |
|  | Reinforcement/ Postpartum Phase  (week 3 postpartum)  -15 minutes | Outcome expectation | - Breastfeeding benefit | WhatsApp follow up  Breastfeeding Support Group |
|  | Reinforcement/ Postpartum Phase  (week 4 postpartum)  -15 minutes | Self-efficacy | -To assess the breastfeeding technique | WhatsApp follow up  Breastfeeding Support Group |
