# Supplementary material for: Effect of Face-to-Face and WhatsApp Communication of a Theory-Based Health Education Intervention on Breastfeeding Self-Efficacy (SeBF Intervention): Cluster Randomized Controlled Field Trial
Source: JMIR Mhealth Uhealth. 2022 Sep 14;10(9):e31996. doi: 10.2196/31996 (PMC9520384; doi:10.2196/31996)
Supplement: Multimedia Appendix 2 [file mhealth_v10i9e31996_app2.pdf]

CONSORT-EHEALTH Checklist V1.6.2 Report	Manuscript Number	31996

(based on CONSORT-EHEALTH V1.6), available at [http://tinyurl.com/consort-ehealth-v1-6].

Date completed

9/17/2021 2:50:52

by

FARAHANA MOHAMAD PILUS

The Effect of Face-To-Face and Whatsapp Communication of Theory-Based Health Education Intervention on Breastfeeding Self-Efficacy: Cluster Randomised Controlled Field Trial

TITLE

1a-i) Identify the mode of delivery in the title

`Whatsapp Communication of Theory-Based Health Education Intervention'

1a-ii) Non-web-based components or important co-interventions in title

`The Effect of Face-To-Face'

1a-iii) Primary condition or target group in the title

`on Breastfeeding Self-Efficacy'

ABSTRACT

1b-i) Key features/functionalities/components of the intervention and comparator in the METHODS section of the ABSTRACT

Social Cognitive Theory was applied in the health education module and delivered via face-to-face and WhatsApp applications.

1b-ii) Level of human involvement in the METHODS section of the ABSTRACT

Involving primigravida or multigravida mothers who did not breastfeed exclusively during their previous pregnancy and reside in a district in Selangor state.

1b-iii) Open vs. closed, web-based (self-assessment) vs. face-to-face assessments in the METHODS section of the ABSTRACT

This study is a two-arm, parallel, single-blind cluster randomized controlled field trial with an intervention and control group. All twelve maternal child health clinics in the district were randomly allocated into six intervention and six control groups. A total of 172 pregnant mothers was randomly assigned to the intervention group (n=82) or control group (n=82). Social Cognitive Theory was applied in the health education module and delivered via face-to-face and WhatsApp applications.

1b-iv) RESULTS section in abstract must contain use data

The rate of attrition was 19.5% (16/82) for control group and 10% (8/82) for intervention group. Using intent-to-treat analysis, the intervention group had a significant increase of breastfeeding self-efficacy mean total score compared to the control group at (F(21, 601) = 111.728, p = <0.001 at eight weeks postdelivery. In addition, the mean total score of breastfeeding knowledge also significantly increased in the intervention group post-intervention compared to the control group at (F(21, 601) = 8.331, p = <0.001. However, no significant difference was noted for the mean total score of breastfeeding attitude post-intervention at (F(21, 602) = 5.502, p = 0.472.

1b-v) CONCLUSIONS/DISCUSSION in abstract for negative trials

Participating in the SeBF programme, a face-to-face and WhatsApp-based programme based on Social Cognitive Theory, enhanced self-efficacy and knowledge about breastfeeding. WhatsApp is an effective tool for supplementing the delivery of interventions aimed at increasing breastfeeding self-efficacy and knowledge. Educational interventions have an impact on the health of pregnant women as well as future generations' health and well-being.

INTRODUCTION

2a-i) Problem and the type of system/solution

Self-efficacy in breastfeeding refers to a woman's confidence in her ability to breastfeed her infant and it is a health behaviour that is associated with a woman's breastfeeding consistency and attitudes toward breastfeeding maintenance. An increase in breastfeeding self-efficacy projected increases in exclusive breastfeeding rates, implying that breastfeeding self-efficacy is a valuable social change theory for explaining breastfeeding rates

2a-ii) Scientific background, rationale: What is known about the (type of) system

By 2025, the World Health Organization (WHO) aimed to achieve a rate of 50% exclusive breastfeeding for infants younger than six months. Malaysia's National Breastfeeding Policy encourages mothers to exclusively breastfeed their infants from birth to six months of age and beyond. Additionally, the National Plan of Action for Nutrition in Malaysia III (NPANM III) for the period 2016-2025 set the goal of exclusively breastfeeding at least 70% of infants aged 0-5.99 months by 2025. The overall prevalence of exclusive breastfeeding in Malaysia was 47.1% (95 % CI: 43.13-51.18).

Does your paper address CONSORT subitem 2b?

The purpose of this study is to determine the effect of a SCT-based intervention namely SeBF (Self-efficacy in breastfeeding) to improve breastfeeding self-efficacy. This intervention was developed to be delivered using face-to-face and WhatsApp communication.

METHODS

3a) CONSORT: Description of trial design (such as parallel, factorial) including allocation ratio

This study is a two-arm, parallel, single-blind cluster randomized controlled field trial with intervention and control groups.

3b) CONSORT: Important changes to methods after trial commencement (such as eligibility criteria), with reasons

Not related in this study

3b-i) Bug fixes, Downtimes, Content Changes

Not applicable in this study.

4a) CONSORT: Eligibility criteria for participants

Pregnant women between 34 and 37 weeks of pregnancy who attended antenatal follow-up at maternal and child health clinics in the Hulu Langat District, Selangor were offered to participate in this study and assessed for eligibility. Eligible mothers were primigravida or multigravida women who had previously failed to exclusively breastfeed during a previous pregnancy and who owned a mobile phone with internet access and WhatsApp application. Mothers who were taking drugs such as anticancer drugs, and mothers who had medical or pregnancy complications that inhibited or complicated breastfeeding (heart disease, cancer, nephritis, active or untreated tuberculosis, HIV or AIDS, active herpes lesions on the breast, and severe malnutrition) were excluded from this study.

4a-i) Computer / Internet literacy

Not mentioned in the study

4a-ii) Open vs. closed, web-based vs. face-to-face assessments:

The SeBF module was developed to be delivered via face-to-face and WhatsApp applications.

4a-iii) Information giving during recruitment

Refer Table 1 for further explaination.

4b) CONSORT: Settings and locations where the data were collected

At each clinic, antenatal mothers who met the eligibility requirements and consented were recruited with an equal number of participants assigned to each clinic.

4b-i) Report if outcomes were (self-)assessed through online questionnaires

Only intervention were on WhatsApp application.

4b-ii) Report how institutional affiliations are displayed

The cluster is maternal and child health clinics. All twelve maternal and child health clinics in Hulu Langat District were randomly allocated to intervention and control groups.

5) CONSORT: Describe the interventions for each group with sufficient details to allow replication, including how and when they were actually administered

5-i) Mention names, credential, affiliations of the developers, sponsors, and owners

Conflicts of interest : None declared.

5-ii) Describe the history/development process

Prior intervention studies served as the basis for the development of the intervention module. These SCT (SCT) based interventions showed to be effective in increasing breastfeeding self-efficacy. This intervention module is a multi-component educational intervention based on SCT principles. Given the relationship between self-efficacy and successful exclusive breastfeeding, a proxy for self-efficacy was used. The combination delivery timeline from antenatal to postnatal and data collection timeline up to eight weeks indicates the justification timeframe for this study. The newly developed intervention module namely "Self-Efficacy in Breastfeeding" (SeBF) module was validated by an expert group comprised of two Public Health Medicine The intervention module also has been tested in a pilot trial with ten mothers who are not participating in the study.

5-iii) Revisions and updating

Not applicable in this study.

5-iv) Quality assurance methods

Were mention in details in the theisis.

5-v) Ensure replicability by publishing the source code, and/or providing screenshots/screen-capture video, and/or providing flowcharts of the algorithms used

Not applicable in the study yet.

5-vi) Digital preservation

Not applicable in the study yet.

5-vii) Access

Through personal WhasApp application.		
**5-viii) Mode of delivery, features/functionalities/components of the intervention and comparator, and the theoretical framework**		
The SeBF module was developed to be delivered via face-to-face and WhatsApp applications. Table 1 explains the contents of the SeBF module and the SCT construct used. There were two phases of the intervention involving the training phase and the reinforcement phase. The training phase was provided during the antenatal period via face-to-face. The reinforcement phase was provided during the antenatal and postnatal period via the WhatsApp application. During the training session, all respondents were introduced to the district's breastfeeding support group and were encouraged to seek their assistance during the intervention period.		
**5-ix) Describe use parameters**		
The face-to-face session involved health educational talk about breastfeeding, practical video demonstration, model demonstration and group discussion which took about 30 minutes to be completed. All the participants in the intervention group were added to a private WhatsApp group after the face-to-face session.<br><br>There were two primary WhatsApp groups consist of antenatal mothers in the training phase and postnatal mothers in the reinforcement phase. The researcher followed up with each participant three times weekly during the antenatal period and four times weekly during the postpartum period by using WhatsApp.		
**5-x) Clarify the level of human involvement**		
Not specifically mentioned in the study,		
**5-xi) Report any prompts/reminders used**		
The researcher followed up with each participant three times weekly during the antenatal period and four times weekly during the postpartum period by using WhatsApp. Important messages and reminders were delivered to each participant using scripts that had been prepared in advance.		
**5-xii) Describe any co-interventions (incl. training/support)**		
Both face-to-face and WhatsApp application are equally important in the intervention.		
**6a) CONSORT: Completely defined pre-specified primary and secondary outcome measures, including how and when they were assessed**		
Baseline data were collected after recruitment. A second assessment was conducted immediately after the training phase, followed by four and eight weeks postpartum. Concurrently, data from the control group were also being collected at the same four time points.		
**6a-i) Online questionnaires: describe if they were validated for online use and apply CHERRIES items to describe how the questionnaires were designed/deployed**		
Not applicable in this study.		
**6a-ii) Describe whether and how "use" (including intensity of use/dosage) was defined/measured/monitored**		
The IBM Statistical Package of Social Sciences System version 25 (SPSS 25) was used for the analyses. Shapiro-Wilk, Komolgorov-Smirnov, and histogram tests have been used to determine normal distribution. The descriptive study presented data in the form of mean, standard deviation, frequency, and percentage. The Pearson Chi-Square/Fisher Exact test was used to determine the homogeneity of baseline data between the intervention and control groups for categorical data and the Student's t-test was used for continuous data. The effectiveness of the intervention was determined using Generalised Mixed Model Analysis (GLMM), which was controlled by baseline covariates such as age, ethnicity, level of education, mother employment, and monthly family income. This study employed a 0.05 level of significance with a 95% confidence interval. Thus, the null hypothesis was rejected with a p-value of 0.05.		
**6a-iii) Describe whether, how, and when qualitative feedback from participants was obtained**		
Not applicable in this study.		
**6b) CONSORT: Any changes to trial outcomes after the trial commenced, with reasons**		
At each clinic, antenatal mothers who met the eligibility requirements and consented were recruited with an equal number of participants assigned to each clinic.		
**7a) CONSORT: How sample size was determined**		
**7a-i) Describe whether and how expected attrition was taken into account when calculating the sample size**		
The final sample size of 160 is based on a 30% increase in breastfeeding self-efficacy in the control group and a 55% increase in breastfeeding self-efficacy in the intervention group, with α of 0.05 and β of 0.20, an intraclass correlation coefficient of 0.05, attrition rate 20% and an average cluster size of 20 with a design effect of 2.45. After adjusting for the clustered design effect, the final sample size required is 160 participants, with 80 participants in each intervention and control group.		
**7b) CONSORT: When applicable, explanation of any interim analyses and stopping guidelines**		
Baseline data were collected after recruitment. A second assessment was conducted immediately after the training phase, followed by four and eight weeks postpartum. Concurrently, data from the control group were also being collected at the same four time points.		
**8a) CONSORT: Method used to generate the random allocation sequence**		
All the clinics were coded and the software Stat Trek was used to perform simple randomization with a 1:1 allocation ratio.		
**8b) CONSORT: Type of randomisation; details of any restriction (such as blocking and block size)**		
All the clinics were coded and the software Stat Trek was used to perform simple randomization with a 1:1 allocation ratio.		
**9) CONSORT: Mechanism used to implement the random allocation sequence (such as sequentially numbered containers), describing any steps taken to conceal the sequence until interventions were assigned**		
The cluster is maternal and child health clinics. All twelve maternal and child health clinics in Hulu Langat District were randomly allocated to intervention and control groups. The allocation was carried out by a nurse who is not involved in this study. All the clinics were coded and the software Stat Trek was used to perform simple randomization with a 1:1 allocation ratio (23). The researcher was only aware of the intervention group's assignment after randomization was completed. At each clinic, antenatal mothers who met the eligibility requirements and consented were recruited with an equal number of participants assigned to each clinic. Mothers were blinded by the group assignment.		
**10) CONSORT: Who generated the random allocation sequence, who enrolled participants, and who assigned participants to interventions**		
The allocation was carried out by a nurse who is not involved in this study. The researcher was only aware of the intervention group's assignment after randomization was completed. At each clinic, antenatal mothers who met the eligibility requirements and consented were recruited with an equal number of participants assigned to each clinic by the researchers. Mothers were blinded by the group assignment.		
**11a) CONSORT: Blinding - If done, who was blinded after assignment to interventions (for example, participants, care providers, those assessing outcomes) and how**		
**11a-i) Specify who was blinded, and who wasn't**		
Participants was blinded in this study.		
**11a-ii) Discuss e.g., whether participants knew which intervention was the "intervention of interest" and which one was the "comparator"**		
Mothers were blinded by the group assignment.		
**11b) CONSORT: If relevant, description of the similarity of interventions**		
Not applicable in this study.		
**12a) CONSORT: Statistical methods used to compare groups for primary and secondary outcomes**		
The effectiveness of the intervention was determined using Generalised Mixed Model Analysis (GLMM), which was controlled by baseline covariates such as age, ethnicity, level of education, mother employment, and monthly family income.		
**12a-i) Imputation techniques to deal with attrition / missing values**		
Not mentioned in the article, was mention clearly in the thesis.		
**12b) CONSORT: Methods for additional analyses, such as subgroup analyses and adjusted analyses**		
Not applicable in this study.		
RESULTS		
**13a) CONSORT: For each group, the numbers of participants who were randomly assigned, received intended treatment, and were analysed for the primary outcome**		
Results show significant difference between intervention and control groups for breastfeeding self-efficacy $F(21, 601) = 111.728$, $p < 0.001$.		
**13b) CONSORT: For each group, losses and exclusions after randomisation, together with reasons**		
Refer Figure 1. in this article.		
**13b-i) Attrition diagram**		
Not mentioned in this articles.		
**14a) CONSORT: Dates defining the periods of recruitment and follow-up**		
Not specifically mentioned in this study.		
**14a-i) Indicate if critical "secular events" fell into the study period**		
Not applicable in this study.		
**14b) CONSORT: Why the trial ended or was stopped (early)**		
Not applicable in this study.		
**15) CONSORT: A table showing baseline demographic and clinical characteristics for each group**		
Was mentioned in detail in the thesis.		
**15-i) Report demographics associated with digital divide issues**		
Not mentioned in the article.		
**16a) CONSORT: For each group, number of participants (denominator) included in each analysis and whether the analysis was by original assigned groups**		

**16-i) Report multiple “denominators” and provide definitions**		
Not mentioned in this study.		
**16-ii) Primary analysis should be intent-to-treat**		
Intention-to-treat were mentioned in the article.		
**17a) CONSORT: For each primary and secondary outcome, results for each group, and the estimated effect size and its precision (such as 95% confidence interval)**		
Table 4 shows the GLMM results for breastfeeding self-efficacy, knowledge and attitude scores after controlling for covariates. Results show significant difference between intervention and control groups for breastfeeding self-efficacy and knowledge; $F(21, 601) = 111.728$, $p < 0.001$ and $F(21, 601) = 8.331$, p = <0.001 respectively. However there is no significant difference between groups for breastfeeding attitude.		
**17a-i) Presentation of process outcomes such as metrics of use and intensity of use**		
Not mentioned in the article		
**17b) CONSORT: For binary outcomes, presentation of both absolute and relative effect sizes is recommended**		
Not applicable in the study.		
**18) CONSORT: Results of any other analyses performed, including subgroup analyses and adjusted analyses, distinguishing pre-specified from exploratory**		
Not mentioned in the article.		
**18-i) Subgroup analysis of comparing only users**		
Not applicable in the study.		
**19) CONSORT: All important harms or unintended effects in each group**		
Was mentioned in the thesis.		
**19-i) Include privacy breaches, technical problems**		
All data relevant to the study are included in the article or uploaded as supplementary information.		
**19-ii) Include qualitative feedback from participants or observations from staff/researchers**		
Not done in the study yet		
DISCUSSION		
**20) CONSORT: Trial limitations, addressing sources of potential bias, imprecision, multiplicity of analyses**		
**20-i) Typical limitations in ehealth trials**		
It is hard to track whether or not the invention groups have read and digested the material distributed in the WhatsApp application.		
**21) CONSORT: Generalisability (external validity, applicability) of the trial findings**		
**21-i) Generalizability to other populations**		
This study also may not be relevant to individuals without access to a smartphone, as intervention follow-up was conducted using the WhatsApp application.		
**21-ii) Discuss if there were elements in the RCT that would be different in a routine application setting**		
Yes, by applying WhatsApp application for follow up.		
**22) CONSORT: Interpretation consistent with results, balancing benefits and harms, and considering other relevant evidence**		
**22-i) Restate study questions and summarize the answers suggested by the data, starting with primary outcomes and process outcomes (use)**		
The purpose of this study is to develop, implement and evaluate the effectiveness of theory-based intervention using SCT on breastfeeding self-efficacy among antenatal mothers in Hulu Langat District, Selangor. The findings show that the intervention group had significantly increased their breastfeeding self-efficacy and knowledge scores compared to the controlled group which is consistent with the previous study in Iraq indicating a significant difference ($p<0.01$) in the change in mean scores between mothers who received the intervention and those who did not receive the intervention by applying SCT in their study.<br><br>These significant changes could be contributed by the use of the SC namely observational learning, personal experience, verbal persuasion, and problem-solving to fulfil the end expectation and breastfeeding self-efficacy. It included an antenatal period that began at the earliest 34 weeks of pregnancy and a postpartum period that lasted until 8 weeks postpartum. This is to ensure that all intervention participants received an adequate and optimal dose of breastfeeding self-efficacy elements during the most critical period, which is from the third trimester of pregnancy to eight weeks postpartum. Numerous approaches, including educational talk, practical breastfeeding video, model demonstrations, and group discussions, all contributed to respondents' increased self-efficacy. The assistance of a breastfeeding support group enhanced respondents' self-efficacy in breastfeeding. The usage of mobile technology, specifically the WhatsApp application, may have improved the primary and secondary outcomes. It facilitated the communication between the researcher and the participants in the intervention group. WhatsApp can speed up new communication and consultations by sending correct and timely information, including legitimate and effective images. Text messaging is a common form of communication among the younger population, and it is the primary mode of communication for the majority of young mothers on a social basis.		
**22-ii) Highlight unanswered new questions, suggest future research**		
In the future, the researcher should give respondents random questions to ascertain their comprehension pertaining to intervention’s material. This study should be prolonged by six months to ensure that all respondents receive appropriate training and follow-up, as well as to determine whether or not this module is capable of attaining the exclusive breastfeeding goals. To teach clients about breastfeeding self-efficacy successfully, all healthcare workers should get adequate breastfeeding self-efficacy training. This module may be used to assist breastfeeding support groups in increasing their program's efficacy.		
Other information		
**23) CONSORT: Registration number and name of trial registry**		
The National Medical Research Registry (NMRR) granted ethical approval for this study (NMRR-19-2712-50586) (IIR). The study was prospectively registered in the Thai Clinical Trial Registry with the identification number TCTR20200213004.		
**24) CONSORT: Where the full trial protocol can be accessed, if available**		
Not applicable in this study		
**25) CONSORT: Sources of funding and other support (such as supply of drugs), role of funders**		
No funding received to conduct this study.		
**X26-i) Comment on ethics committee approval**		
The National Medical Research Registry (NMRR) granted ethical approval for this study (NMRR-19-2712-50586) (IIR). Each respondent provided written and informed consent during the data collection process. All participants’ information was kept strictly confidential. The study was prospectively registered in the Thai Clinical Trial Registry with the identification number TCTR20200213004.		
**x26-ii) Outline informed consent procedures**		
Not mentioned in the articles.		
**X26-iii) Safety and security procedures**		
Not mentioned in the articles.		
**X27-i) State the relation of the study team towards the system being evaluated**		
Conflicts of interest : None declared.		
